# Construction of a co-expression network affecting intramuscular fat content and meat color redness based on transcriptome analysis

**DOI:** 10.3389/fgene.2024.1351429

**Published:** 2024-02-13

**Authors:** Binbin Wang, Liming Hou, Wen Yang, Xiaoming Men, Keke Qi, Ziwei Xu, Wangjun Wu

**Affiliations:** ^1^ Institute of Animal Husbandry and Veterinary, Zhejiang Academy of Agricultural Sciences, Hangzhou, China; ^2^ College of Animal Science and Technology, Nanjing Agricultural University, Nanjing, China

**Keywords:** intramuscular fat content, meat color redness, RNA-seq, functional enrichment analysis, hub gene

## Abstract

**Introduction:** Intramuscular fat content (IFC) and meat color are vital indicators of pork quality.

**Methods:** A significant positive correlation between IFC and redness of meat color (CIE a* value) indicates that these two traits are likely to be regulated by shared molecular pathways.To identify candidate genes, hub genes, and signaling pathways that regulate these two traits, we measured the IFC and CIE a* value in 147 hybrid pigs, and selected individuls with extreme phenotypes for transcriptome analysis.

**Results:** The results revealed 485 and 394 overlapping differentially expressed genes (DEGs), using the DESeq2, limma, and edgeR packages, affecting the IFC and CIE a* value, respectively. Weighted gene co-expression network analysis (WGCNA) identified four modules significantly correlated with the IFC and CIE a* value. Moreover, we integrated functional enrichment analysis results based on DEGs, GSEA, and WGCNA conditions to identify candidate genes, and identified 47 and 53 candidate genes affecting the IFC and CIE a* value, respectively. The protein protein interaction (PPI) network analysis of candidate genes showed that 5 and 13 hub genes affect the IFC and CIE a* value, respectively. These genes mainly participate in various pathways related to lipid metabolism and redox reactions. Notably, four crucial hub genes (*MYC, SOX9, CEBPB, and PPAGRC1A*) were shared for these two traits.

**Discussion and conclusion:** After functional annotation of these four hub genes, we hypothesized that the *SOX9/CEBPB/PPARGC1A* axis could co-regulate lipid metabolism and the myoglobin redox response. Further research on these hub genes, especially the *SOX9/CEBPB/PPARGC1A* axis, will help to understand the molecular mechanism of the co-regulation of the IFC and CIE a* value, which will provide a theoretical basis for improving pork quality.

## 1 Introduction

Pork is a significant and extensively utilized animal resource that has emerged as a principal protein source within human diets. In recent years, China’s yearly pork production has surpassed 50 million tons. Duroc × (Landrace × Yorkshire) (DLY) pigs account for over 90% of the pork market due to their rapid growth and high lean meat rate (Duan et al., 2023). With improved living standards, high-quality pork has become more popular among consumers. Meat quality is a crucial indicator for assessing pork production and quality. Essential indicators of meat quality include intramuscular fat content (IFC), meat color, tenderness, and drip loss, which can directly impact pork quality and market competitiveness (Moeller et al., 2010). Consumers favor snowflake meat (a reflection of high IFC or marbling), and IFC deposition is the main cause of snowflake meat (Liu et al., 2020). Meat color is also one of the most direct sensory indicators of pork quality for consumers and directly affects their consumption behavior. In the food industry, the most popular numerical colour space system is the L* (lightness), b* (yellowness) and a* (redness), which is also referred as the CIELAB system, originally defined by the CIE (CIE, 1986). The subjective color scores of the meat showed a stronger correlation with the CIE a* value (*R* = 0.80) in one study (Sun et al., 2016). Hence, the quality of pork color could be directly assessed based on the CIE a* value. Despite DLY pork effectively meeting the quantitative demand, its muscle quality falls short of eliciting satisfaction. Both the IFC and CIE a* value are traits with relatively high heritability (Cabling et al., 2015; Wang et al., 2022) and are the most intuitive indicators of high-quality pork. Consequently, increasing the IFC and CIE a* value through genetic improvement is a major research focal point for pig breeding enterprises.

IFC refers to the amount of fat that accumulates between muscle fibers or within muscle cells, mainly composed of phospholipids and triglycerides (Shi-Zheng and Su-Mei, 2009). It is widely accepted that changes in meat color in muscles are due to changes in myoglobin levels. This may be due to higher myoglobin levels in slow/oxidative myofibers (red muscle fibers) than in fast/glycolytic myofibers (white muscle fibers). When there is a high proportion of red muscle fibers in muscle tissue, its muscle color exhibits a more distinct red characteristic (Kim et al., 2010). This phenomenon is closely related to the biochemical markers of meat, such as the oxidation state, cytochrome content, and redox forms. Previous studies have shown a significant correlation (*R* = 0.260–0.323) between IFC and CIE a* (Mortimer et al., 2014; Zhang et al., 2022). Therefore, we speculated that these two traits might have similar genetic backgrounds, but the underlying genetic basis was largely unknown.

Differences in phenotype are caused by a variety of factors, among which changes in gene expression are crucial. Therefore, the variations in the IFC and CIE a* value within a population might be driven by differences in the expression levels of critical genes involved in regulating these two traits. With the development of next-generation sequencing technologies, the emergence of transcriptome sequencing (RNA-seq) allowed us to detect the expression levels of all genes across the entire genome. Researchers usually use individuals with extreme phenotypes of the IFC and *a* * value to perform RNA-seq, allowing them to obtain many candidate genes and signaling pathways related to the IFC and CIE a* value (Cardoso et al., 2017; Xing et al., 2021; Fernndez-Barroso et al., 2022).

However, organisms are complex systems with interconnected genes regulating biological activities, forming intricate network systems. Therefore, it is crucial to consider the interrelationships between thousands of genes when studying phenotypic variation. Differential expression analysis may not capture critical biological pathways or gene-gene interactions relevant to target traits, as it focuses on the impact of individual genes rather than the influence of gene networks (Xing et al., 2021). Coexpressed genes often form densely connected subgraphs in networks, representing functionally related gene groups or signaling pathways, and exhibit specific biological functions by developing local substructure modules (Barabasi and Oltvai, 2004). These modules reveal interactions among genes at a systems level, aiding researchers in further understanding the mechanisms underlying gene interactions and identifying regulatory hubs of coexpressed genes (Talukdar et al., 2016). Weighted gene co-expression network analysis (WGCNA) is an efficient and accurate method for describing the correlation among all genes or modules within the whole genome with traits. It is particularly advantageous for simultaneously identifying key genes of multiple complex traits (Zhang and Horvath, 2005), such as fat deposition (Xing et al., 2021), meat quality (Zhao et al., 2020), and reproductive performance (Wu et al., 2022).

Based on transcriptomic data, the present study aimed to gain molecular insights into the hub genes and metabolic pathways that coregulate the variations in the IFC and CIE a* value. We collected individuals with divergent IFC and CIE a* values for RNA-seq. Subsequently, we identified the differentially expressed genes (DEGs), and performed gene set enrichment analysis (GSEA), WGCNA, and protein protein interaction (PPI) analysis. We identified the candidate genes and modules significantly related to these two traits. Through systematic integration of the above results, we identified the hub genes and pathways that could co-regulate the changes in the IFC and CIE a* values. These findings contribute to understanding the genetic mechanisms of co-regulation changes in the IFC and CIE a* value. Moreover, the identified hub genes may serve as potential biomarkers for the synergistic improvement of IFC and meat color in pigs.

## 2 Materials and methods

### 2.1 Animals, sample collection, and phenotype measurement

A total of 147 commercial DLY pigs, consisting of 70 castrated boars and 77 females, were selected for this study. The experimental pigs were reared under standardized indoor conditions and provided *ad libitum* access to feed and water at Jiangsu Kangle Pig Breeding Farm (Changzhou, China). All experimental protocols involving animals were approved by the Nanjing Agricultural University Animal Care and Use Committee (Certification No.: SYXK (Su) 2022–0031). These pigs were slaughtered in six batches at the same slaughterhouse within a month, with 20–30 pigs slaughtered in each batch, with an average live weight of 122.49 ± 16.54 kg (mean ± standard deviation). Following slaughter, LD muscle from the last third and fourth thoracic vertebrae was collected for each pig. Approximately 0.5 g of LD muscle was placed into a 1.5 mL tube and frozen at −80 °C for RNA extraction. Another portion of LD muscle was trimmed to 1 cm × 1 cm × 2 cm along the fiber direction and fixed in 4% paraformaldehyde solution. The meat color redness value of the LD muscle was assessed three times at 24 h post-mortem using a CR-410 hand-held colorimeter (Kinica Minolta Sensing Inc., Shanghai, China). The mean of the three measurements was the final CIE a* value. Approximately 300 g of LD muscle was utilized for determining IFC using the Soxhlet extraction method (Supakankul and Mekchay, 2016).

### 2.2 Sample selection

In order to avoid the influence of sex and carcass weight on the selected samples, a general linear model in SAS software was used to analyze the factors affecting the IFC and redness values in 147 DLY pigs. The results showed that sex and carcass weight did not affect IFC and CIE a* values. Therefore, based on the extreme values of IFC and CIE a* values, we selected the high IFC group (H_IFC, n = 6), low IFC group (L_IFC, n = 6), high CIE a* group (H_a*, n = 6), and low CIE a* group (L_a*, n = 6), respectively. During the selection process, we found that there were 2 samples overlapping between the H_IFC group and the H_a* group, and 3 samples overlapping between the L_IFC group and the L_a* group. So, 19 unique samples were used for transcriptome analysis in this study. The means of the IFC and CIE a* value in the high and low groups were calculated using the two-tailed Student’s t-test. Besides, we also calculated the differences of the samples in the H_IFC and H_a* value groups (H_group, n = 10) and the samples in the L_IFC and L_a* groups (L_group, n = 9) using the two-tailed Student’s t-test. All analyses were conducted using SPSS (v22.0) software (SPSS Inc., Chicago, IL, United States).

### 2.3 Haematoxylin–eosin staining

Selected LD samples were fixed in 4% paraformaldehyde for 24 h at room temperature. Muscle tissue was dehydrated using ethanol, transparently treated with xylene, embedded in paraffin, and cut into 3–4 μm samples for further haematoxylin–eosin (H&E) staining. Sections were deparaffinized in xylene, rehydrated in ethanol and stained with hematoxylin for 10 min. The sections were then rinsed in tap water and stained with eosin for 1 min, dehydrated, transparently treated with xylene and finally sectioned using neutral gum. The prepared sections were observed under the microscope, in which the nuclei and cytoplasm of the muscle cells appeared blue and light red, respectively, and the adipocytes appeared white.

### 2.4 RNA extraction, library construction, and sequencing

Total RNA was extracted from 100 mg of frozen LD muscle using TRIzol reagent (Invitrogen, Carlsbad, CA, United States). The total RNA was quantified and quality controlled using Qubit 2.0 and Agilent 2,100. RNA with an RNA integrity number (RIN) of >7 and RNA quality rating of “A” was used for RNA library construction. RNA libraries were constructed using the VAHTS^®^ universal V8 RNA-seq Library Prep Kit for Illumina (Vazyme, China) according to the manufacturer’s instructions. The Illumina NovaSeq 6,000 platform (Illumina, San Diego, CA, United States) was used for transcriptome sequencing based on the high-quality RNA library, and the sequencing read length was paired-end 150 bp. The obtained raw data were filtered to clean data with FastQC (v0.11.5) and Trimmomatic (v0.38) software (Bolger et al., 2014) by removing reads containing adapters, low-quality reads, and reads with an N content of >5%. The sequencing depth of transcriptome data in this study exceeded 40 million reads per sample. The average sequencing depth of the clean reads used for subsequent analysis was 42.91 million reads. The alignment analysis results showed that the average unique mapping rate was 87.53%. The clustering heatmaps between samples showed significant stratification between high and low groups (Supplementary Figure S1). Overall, the sequencing data exhibited high quality, rendering it suitable for subsequent analyses.

### 2.5 Identification of DEGs

The obtained clean reads were mapped to the *Sus scrofa* 11.1 genome from Ensembl 101 using STAR (v2.7.2) software (Dobin et al., 2013) with settings (–sjdbOverhang 135). Finally, a transcriptome gene expression count file was converted using featureCounts (v2.0.0) software (Liao et al., 2014). The DESeq2 (v1.25.9) (Love et al., 2014), limma (Ritchie et al., 2015), and edgeR packages in R (v4.1) (Robinson et al., 2010) software were used to identify DEGs between the groups. DEGs were defined as those with a false discovery rate (FDR) of <0.05 and |log2FoldChange| ≥ 1. Furthermore, overlapping DEGs detected by the DESeq2, limma, and edgeR packages were considered true DEGs, and used for subsequent functional enrichment analysis.

### 2.6 Functional annotation and enrichment analysis

To better understand the functions of overlapping DEGs, the R package BioMart (Haider et al., 2009) was used to annotate genes using the reference genome *Sus scrofa* 11.1. The Gene Ontology (GO) terms and Kyoto Encyclopedia of Genes and Genomes (KEGG) pathways of overlapping DEGs were subjected to functional enrichment analysis using the R package clusterProfiler (v4.6.2) (Wu et al., 2021) with the following default parameters: ont = “ALL”, nPerm = 1,000, pAdjustMethod = “BH”, minGSSize = 10, maxGSSize = 500. In addition, we removed redundancy from the GO terms using the ‘simplify’ function in the clusterProfiler package, with the following default parameters: cutoff = 0.7, by = “p.adjust”, select_fun = min. The overlapping DEGs were visualized as a heatmap plot using the R function heatmap. Additionally, considering that GSEA does not require an arbitrary cutoff for differential gene expression and has a more extensive functional range, we also used GSEA on our datasets based on whole genes of the IFC and CIE a* groups, using the clusterProfiler package (v4.6.2) (Wu et al., 2021) with the above default parameters. The threshold of significantly enriched GO terms and KEGG pathways was a *q* value of <0.10.

### 2.7 WGCNA

To construct a co-expression network, we used WGCNA, a package from R (1.72.1) (Langfelder and Horvath, 2008), with RNA-seq data (n = 19), with their counts normalized by transcript per million (TPM). After the expression matrix input, genes with TPM values of >1 in more than 10 individuals were selected for a coexpression network setting. The clean expression matrix underwent hierarchical clustering using the group average method to identify outliers, which were samples deviating significantly from the others. There were no outliers in this study, and the final expression matrix contained 10,512 genes and 19 individuals for establishing an unsigned coexpression network based on the step-by-step method.

This study selected a power value of 18 based on the scale-free topology criterion, resulting in a scale-free topology index (*R* ^*2*^) of 0.90. The hybrid dynamic tree-cutting approach employs a minimum module size of 30 as the default and commonly used value. To characterize the module expression, module eigengenes (MEs) were calculated as the first principal component of the expression matrix. The WGCNA approach facilitates the identification of biologically significant modules and potential critical modules for further analysis by defining the module trait relationships (MTRs) and gene significance (GS) of each module. The mean value of GS for the genes within a module represented the module significance (MS). To select candidate modules for functional enrichment analysis, modules with MTRs greater than 0.35 and MS exceeding 0.25 were considered based on the criteria reported in previous studies. The GO and KEGG pathway terms of all genes within the critical module were subjected to functional enrichment analysis using the clusterProfiler package (v4.6.2) (Wu et al., 2021) with the above default parameters.

### 2.8 Identification of candidate and hub genes related to the IFC and CIE a* value

To further identify candidate genes affecting the IFC and CIE a* value, we performed overlap analysis of significantly enriched GO terms and KEGG pathways in Omicshare platform (https://www.omicshare.com/) derived from overlapping DEGs, GESA, and WGCNA, respectively. The results of the overlap analysis are presented in the Venn network diagram. The selected GO terms and KEGG pathways had a *q* value of <0.1 in all three methods and less than 0.05 in at least two methods. DEGs located in the overlapping GO terms and KEGG pathways were considered candidate genes and used for subsequent PPI analysis.

The construction of a PPI network was employed to analyze the interactions between genes encoding proteins in candidate genes based on the Search Tool for the Retrieval of Interacting Genes (STRING) database (v11.5) (Szklarczyk et al., 2015). Cytoscape software (v3.8.0) (Shannon et al., 2003) was employed to visualize the entire PPI network. This analysis allowed the connection patterns between genes in PPI networks to be explored and visualized. Highly connected genes, also known as hub genes, may play an essential role in influencing the target traits of these candidate genes. The criterion for selecting the hub gene was that the degree of connectivity was greater than 10.

## 3 Results

### 3.1 Phenotypes and sequencing data

**FIGURE 1:**
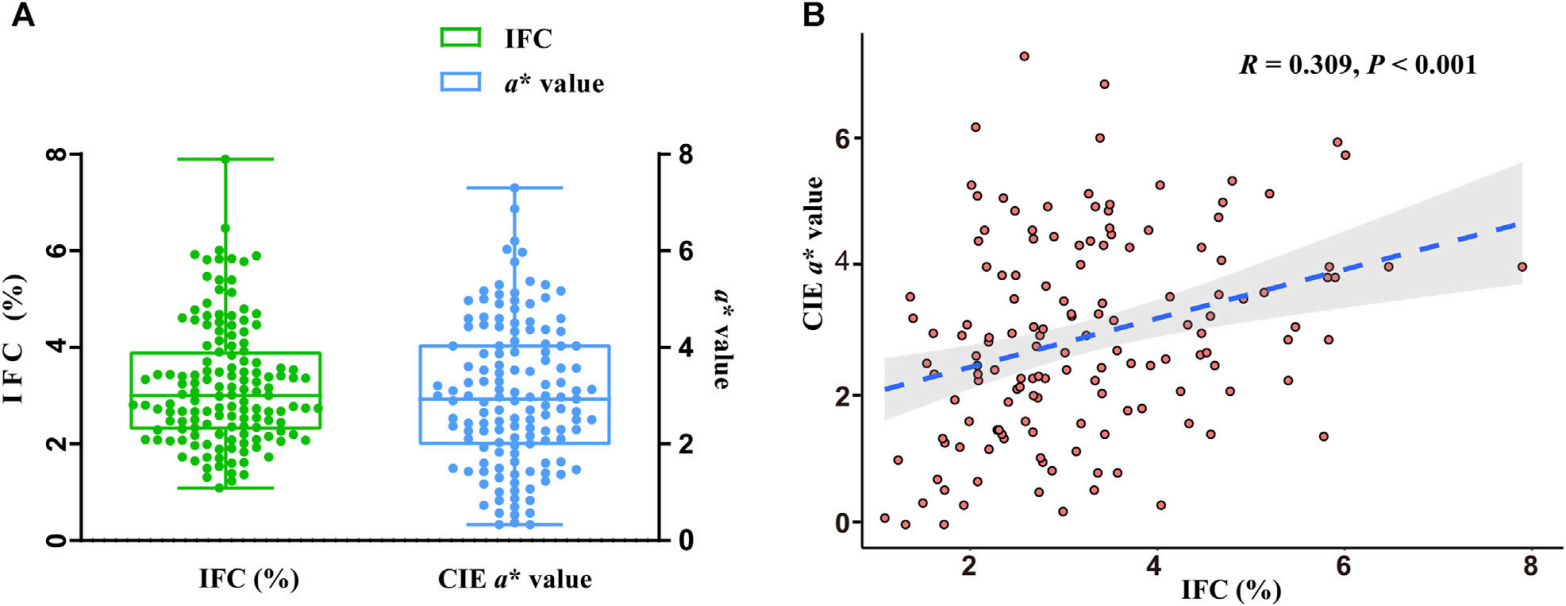
Statistical analysis of intramuscular fat content (IFC) and meat color redness (CIE a*) values. **(A)** Phenotypic values of the IFC and CIE a* value. **(B)** Correlation analysis of the IFC and CIE a* value.

The phenotypes of the IFC and CIE a* value in 147 DLY pigs are shown in Figure 1A. The mean and standard error of the IFC and CIE a* value were 3.20% ± 0.10% and 2.86% ± 0.13%, respectively. The IFC and CIE a* value showed a significant positive correlation in 147 DLY pigs (*R* = 0.309, *p* < 0.001) (Figure 1B).

**FIGURE 2:**
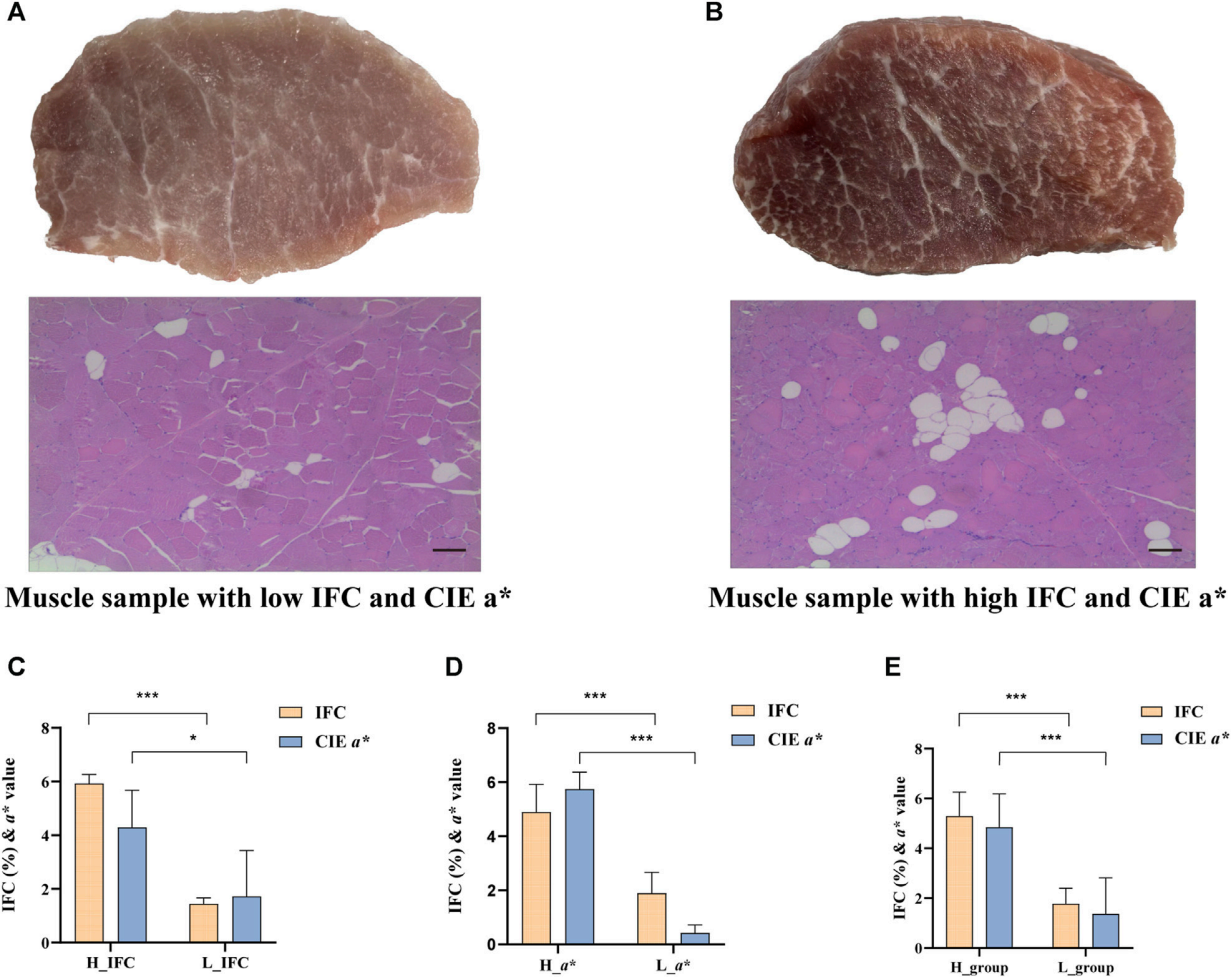
IFC and CIE CIE a* value comparison between the high and low groups. **(A)** Representative plots of latissimus dorsi (LD) tissue and H&E staining of shared samples from the low IFC group and low CIE a* group, scale bar = 100 μm. **(B)** Representative plots of LD tissue and H&E staining of shared samples from the high IFC group and high CIE a* group, scale bar = 100 μm. **(C**–**E)** Comparasion of the IFC and CIE a* value in different groups. The H_group represents the sample combination of H_IFC and H_a*, n = 10; The L_group represents the sample combination of the L_IFC and L_a* value group, n = 9. Error bars represent the standard deviation (SD), where yellow bars represent the IFC and blue bars represent the CIE a* value. *p < 0.05, **p < 0.01, ***p < 0.001, two-tailed Student’s t-test.

Based on the IFC and CIE a* value, the LD muscle samples were divided into the high IFC (H_IFC, n = 6), low IFC (L_IFC, n = 6), high CIE a* value (H_a*, n = 6), and low CIE a* value (L_a*, n = 6) groups. The phenotypic values of selected individuals are shown in Figure 2 and Supplementary Table S1. The mean IFCs of the high and low groups were 5.92% and 1.45%, respectively. The mean CIE a* values of the high and low groups were 4.30 and 1.72, respectively. The IFC and CIE a* value in the high groups (H_IFC and H_a*) were significantly higher than in the low groups (L_IFC and L_a*) (Figures 2C, D). Moreover, the phenotypic information of the samples in the H_IFC and H_a* groups (H_group, n = 10) and the samples in the L_IFC and L_a* groups (L_group, n = 9) was counted, and the results showed that the IFC and CIE a* values in the H_group were 5.30% ± 0.91% and 4.85 ± 1.27, respectively, and were 1.75% ± 0.61% and 1.37 ± 1.37, respectively, in the L_group. The IFC and CIE a* values were significantly higher in the H_group than in the L-group (Figure 2E). In addition, the results of general linear model analysis indicated that sex and carcass weight had no significant impact on the IFC and CIE a* values (Table 1).

**TABLE 1:** Influencing factors of intramuscular fat content (IFC) and redness value in 147 DLY pigs.

Trait	IFC	CIE a* value
Sex	NS	NS
Carcass weight	NS	NS

NS, not significant.

Concerning the RNA-Seq data, 37.48–50.63 million raw reads per sample were generated. After filtering approximately 1.39% of the raw reads, an average of 42.91 million clean reads were used for the following analysis. The mean Q30 and GC percentage values of these clean data were 95.19% and 52.53%, respectively. After alignment using STAR software, 87.53% of the clean reads were uniquely mapped to the *Sus scrofa* 11.1 genome (Supplementary Table S1). Before DEG detection, low expression levels or non-expressed genes were removed based on gene expression counts. The remaining 16,453 genes for IFC and 16,249 for CIE a* were analyzed in the differential expression analysis.

### 3.2 DEGs

**FIGURE 3:**
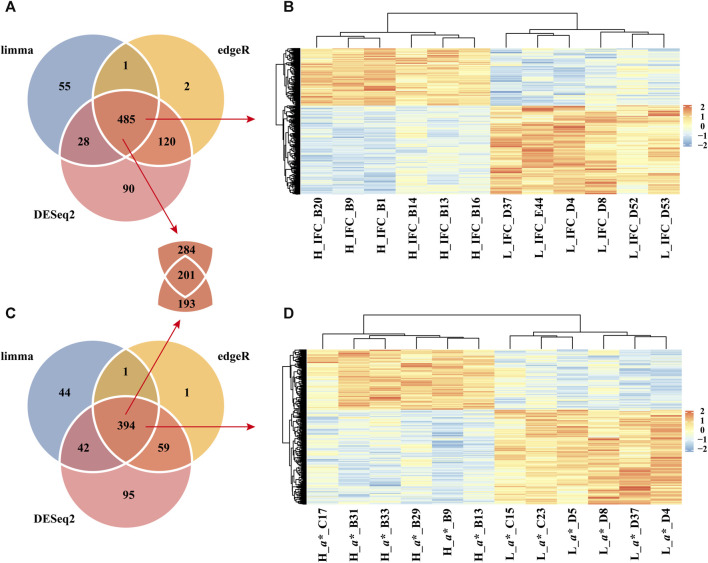
Identification of differentially expressed genes (DEGs). **(A)** Venn diagram of DEGs identified using the DESeq2, limma, and edgeR packages for the IFC. **(B)** Heatmap of overlapping DEGs between the H_IFC and L_IFC groups. **(C)** Venn diagram of DEGs identified using the DESeq2, limma, and edgeR packages for the CIE a* value. **(D)** Heatmap of overlapping DEGs between the H_a* and L_a* groups.

The present study identified 723, 569, and 608 DEGs between the H_IFC and L_IFC groups using DESeq2, limma, and edgeR, respectively (Figure 3A). A total of 485 overlapping DEGs were detected, including 190 upregulated and 295 downregulated DEGs in the H_IFC group, respectively. For the CIE a* value, 590, 481, and 455 DEGs were identified using DESeq2, limma, and edgeR, respectively (Figure 3C). Three hundred and ninety-four DEGs were shared among the three methods, including 153 upregulated and 241 downregulated DEGs in the H_CIE a* group. Figures 3B,D exhibit the heatmap of these overlapping DEGs, from which it can be seen that the expression patterns of overlapping DEGs were consistent within groups and different between groups. Moreover, 201 DEGs were shared between these two traits.

### 3.3 Functional enrichment analysis

**FIGURE 4:**
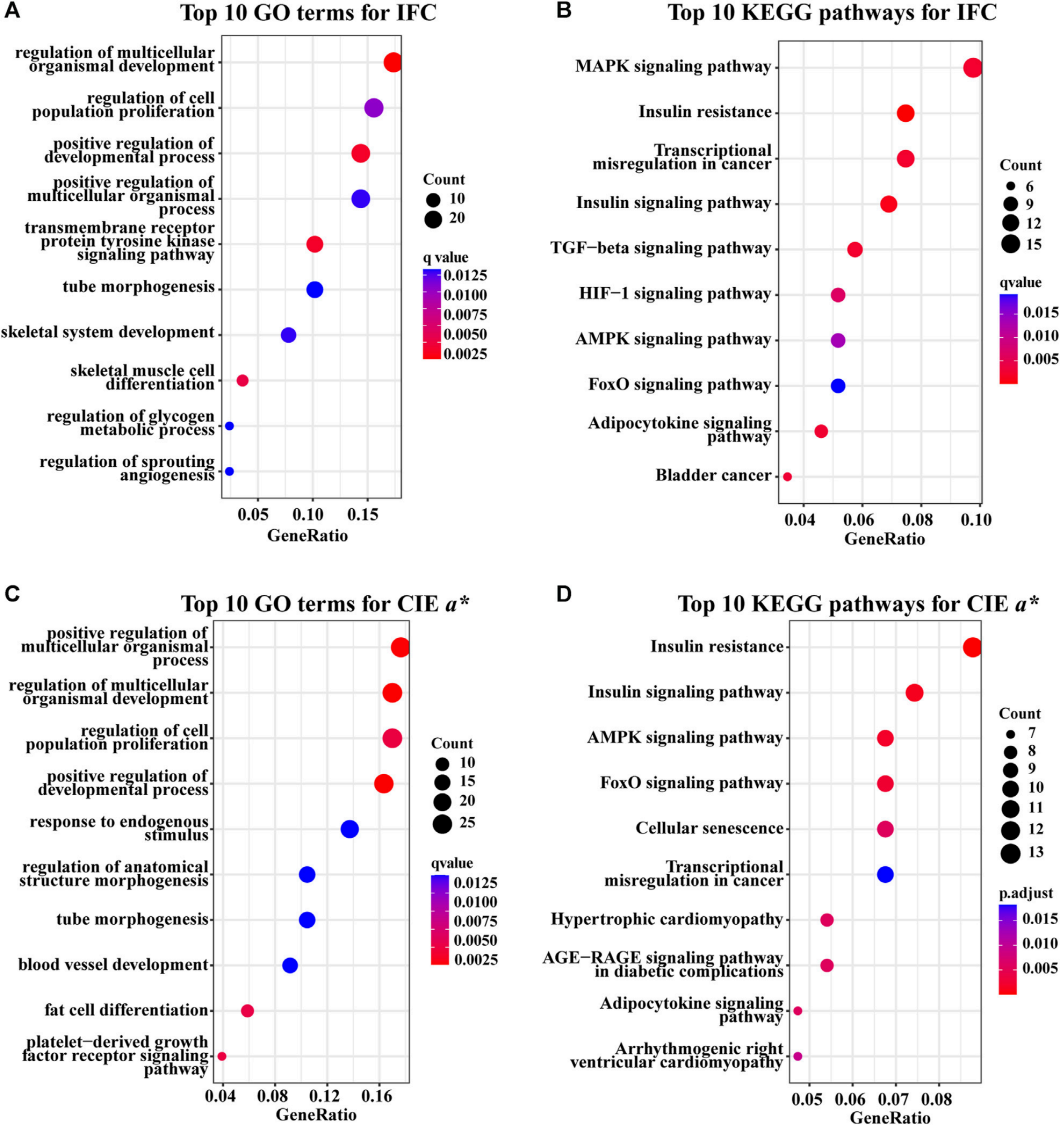
GO and KEGG enrichment analysis of overlapping DEGs. **(A)** Top five GO terms of overlapping DEGs for the IFC in the BP, CC, and MF categories. **(B)** Significantly enriched KEGG pathways of overlapping DEGs for IFC. **(C)** Top five GO terms of overlapping DEGs for CIE a* value in the BP, CC, and MF categories. **(D)** Significantly enriched KEGG pathways of overlapping DEGs for the CIE a* value. The size of the dot represents the number of overlapping DEGs enriched to this GO term or pathway. The colour of the dot represents the significance of the enrichment, where a redder dot indicates greater significance.

There were 106 significantly enriched GO (GO_DEGs) terms (Supplementary Table S4; Figure 4A) and 20 significantly enriched KEGG (KEGG_DEGs) pathways (Supplementary Table S5; Figure 4B) based on overlapping DEGs between the H_IFC and L_IFC groups. Among these 106 enriched GO_DEGs terms, most belonged to the biological process (BP) category, and only 1 and 6 terms belonged to the cellular component (CC) and molecular function (MF) categories, respectively. In terms of KEGG_DEGs pathways, more than half of the 20 significantly enriched pathways were closely associated with lipid metabolism and lipolysis, such as the adipocytokine signaling pathway (ssc04920), MAPK signaling pathway (ssc04010), PI3K-Akt signaling pathway (ssc04151) and regulation of lipolysis in adipocytes (ssc04923). For the CIE a* value, 138 significantly enriched GO_DEGs terms (Supplementary Table S6; Figure 4C) and 22 significantly enriched KEGG_DEGs pathways (Supplementary Table S7; Figure 4D) were detected. Similarly, most of these enriched GO_DEGs terms belonged to the BP category. KEGG_DEGs enrichment analysis revealed that 9 of 12 significant pathways were strongly associated with redox and antioxidant responses, such as the insulin signaling pathway (ssc04910), AMPK signaling pathway (ssc04152), FoxO signaling pathway (ssc04068), adipocytokine signaling pathway (ssc04920), and MAPK signaling pathway (ssc04010). Furthermore, 12 of these 22 significantly enriched pathways were shared with the significantly enriched pathways found in the IFC group. This suggests that there was some similarity in the genetic background between the IFC and CIE a* value.

To further understand the mechanisms of genetic differences between the high and low groups, GSEA was used. The results showed that 168 significantly enriched GO_GSEA terms (Supplementary Table S8) and 61 significantly enriched KEGG_GSEA pathways (Supplementary Table S9) were identified between the H_IFC and L_IFC groups. Among these enriched GO_GSEA terms, the top five were related to mitochondrial metabolism and organismal oxidoreductase activity. In terms of KEGG_GSEA, several significant pathways associated with lipid and fatty acid metabolism were enriched, such as oxidative phosphorylation (ssc00190), fatty acid metabolism (ssc01212), the adipocytokine signaling pathway (ssc04920), and ether lipid metabolism (ssc00565). For the CIE a* value, 390 significantly enriched GO_GSEA terms Supplementary Table S10) and 76 significantly enriched KEGG_GSEA pathways (Supplementary Table S11) were identified between the H_a* and L_a* groups. Redox reactions are an essential factor influencing the CIE a* value; the top five significantly enriched GO_GSEA terms were mainly related to the cellular response to an organic substance, oxidoreductase activity, and positive regulation of the developmental process. KEGG_GSEA results showed that more than 60% of the significantly enriched pathways in the H_a* and L_a* groups were consistent with those significantly enriched in the high and low IFC groups. These overlapping pathways included the above-mentioned lipid metabolic pathways, such as ssc00190, ssc01212, and ssc00565. These results suggested that lipid and fatty acid metabolism are essential factors influencing changes in the CIE a* value.

### 3.4 Co-expressed gene modules associated with the IFC and CIE a* value

**FIGURE 5:**
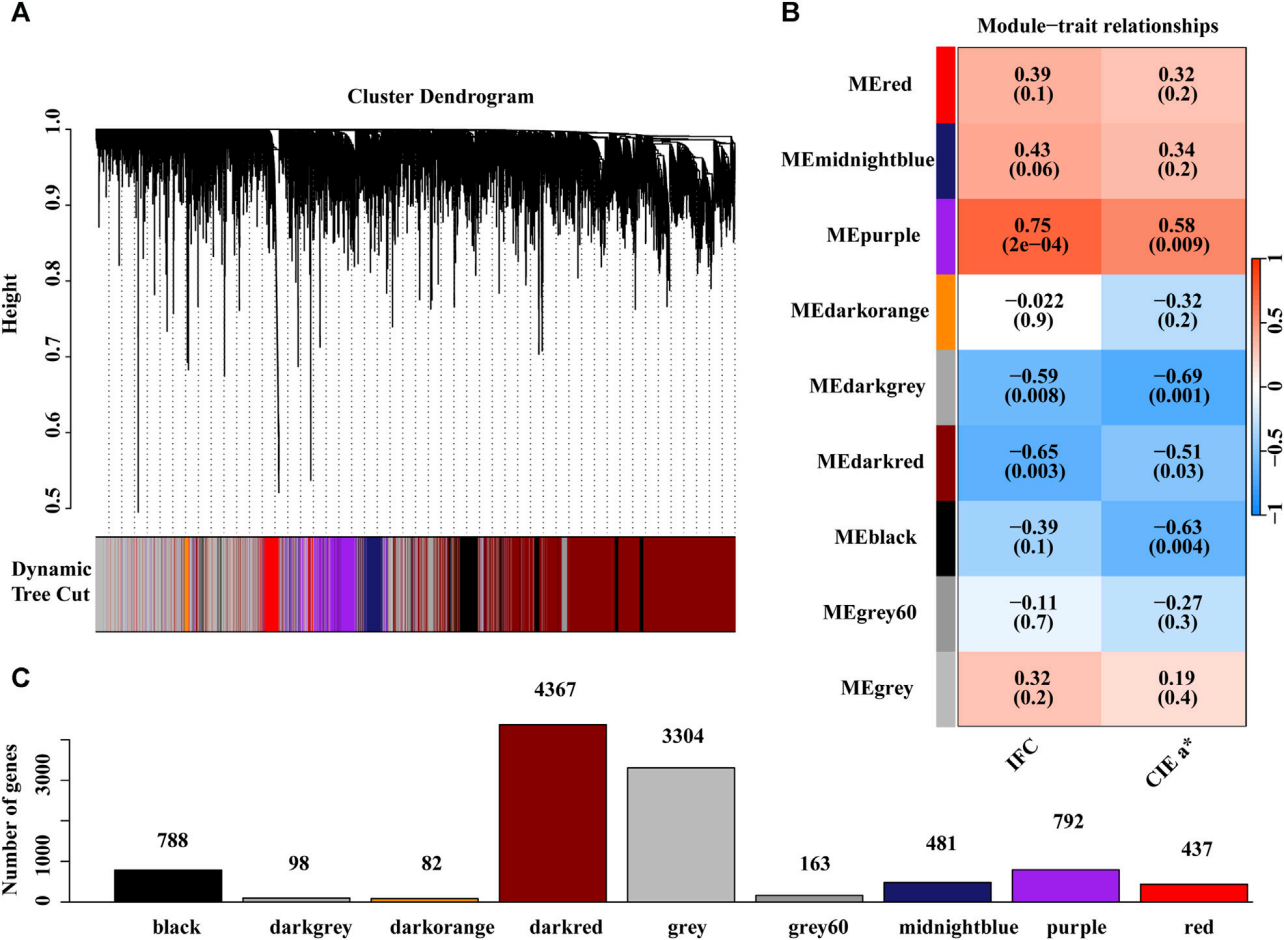
Weighted gene co-expression network analysis (WGCNA). **(A)** The gene dendrogram was obtained by clustering the dissimilarity based on consensus Topological Overlap with the corresponding module colors indicated by the color row. **(B)** Matrix with module trait relationships (MTRs) and corresponding *p* values between the detected modules on the *y*-axis and traits (IFC and CIE a* value) on the *x*-axis, where blue represents a negative correlation, red represents a positive correlation, and white represents no correlation. **(C)** The number of genes contained in each module.

The expression matrix containing 10,512 genes from 19 individuals was used for WGCNA. Hierarchical cluster analysis revealed no outliers among the19 samples (Supplementary Figure S2A). To build a scale-free network, we chose a soft threshold of = 18, with a scale-free topology fitting index *R* ^2^ of >0.90 (Supplementary Figure S2B). In this study, nine gene coexpression modules were identified (Figure 5A). The module with the minimum number of genes among these modules was the dark orange module, containing 82 genes, while the maximum number of genes was in the dark red module, including 4,367 genes (Figure 5B). Correlation analysis between module eigengene and the IFC or CIE a* value was performed, and four modules, including purple, dark grey, dark red, and black, were significantly correlated with the IFC and CIE a* value (Figure 5C; Supplementary Figure S3; Supplementary Figure S4). Among these four significant modules, the purple module positively correlated with both the IFC and CIE a* value. In contrast, the dark grey, dark red, and black modules exhibited negative correlations with the IFC and CIE a* value. These four modules contained a total of 6,045 genes encoding proteins. Subsequently, we focused on 6,045 genes for subsequent functional enrichment analysis. Details of the 6,045 genes are shown in Supplementary Table S12.

### 3.5 Functional enrichment analysis for the four key modules

The significant GO_WGCNA terms and KEGG_WGCNA pathways are presented in Supplementary Tables S13 and S14. The GO_WGCNA results showed that genes in the black red module were significantly enriched in 35 GO terms, which were mainly related to IFC and CIE a*, such as regulation of the catabolic process (GO:0009894), RNA binding (GO:0003723), negative regulation of lipid localization (GO:1905953), and oxidoreduction-driven active transmembrane transporter activity (GO:0015453). From the KEGG_WGCNA analysis results, 156 pathways were significantly enriched, and most of the significant pathways were related to lipid deposition, decomposition, and oxidation-reduction reactions. These pathways are critical in the regulation of both IFC and CIE a*, such as the adipocytokine signaling pathway (ssc04920), FoxO signaling pathway (ssc04068), MAPK signaling pathway (ssc04010), and oxidative phosphorylation (ssc00190).

In the black module, the functional enrichment results showed that 41 GO_WGCNA terms and 86 pathways were significantly enriched. These significant GO_WGCNA terms were mainly involved in phosphorylation (GO:0016310), response to oxygen-containing compounds (GO:1901700), the actin cytoskeleton (GO:0015629), and calcium ion binding (GO:0005509). The significant pathways related to lipid metabolism and oxidative reactions mainly included regulation of lipolysis in adipocytes (ssc04923), glycerolipid metabolism (ssc00561), the PI3K-Akt signaling pathway (ssc04151), the MAPK signaling pathway (ssc04010), and the Wnt signaling pathway (ssc04310).

Genes in the purple module were significantly enriched with 6 GO_WGCNA terms and 14 KEGG_WGCNA pathways. These GO terms were mainly involved in extracellular matrix organization (GO:0030198) and collagen binding (GO:0005518). Among the significant KEGG_WGCNA pathways, four were associated with IFC, such as fatty acid metabolism (ssc01212), insulin resistance (ssc04931), calcium signaling pathway (ssc04020), and fatty acid degradation (ssc00071). Genes in the black grey modules were not significantly enriched in GO terms and KEGG pathways, which might have been due to the limited number of genes in this module.

### 3.6 Identification of candidate genes related to the IFC and CIE a* value

**FIGURE 6:**
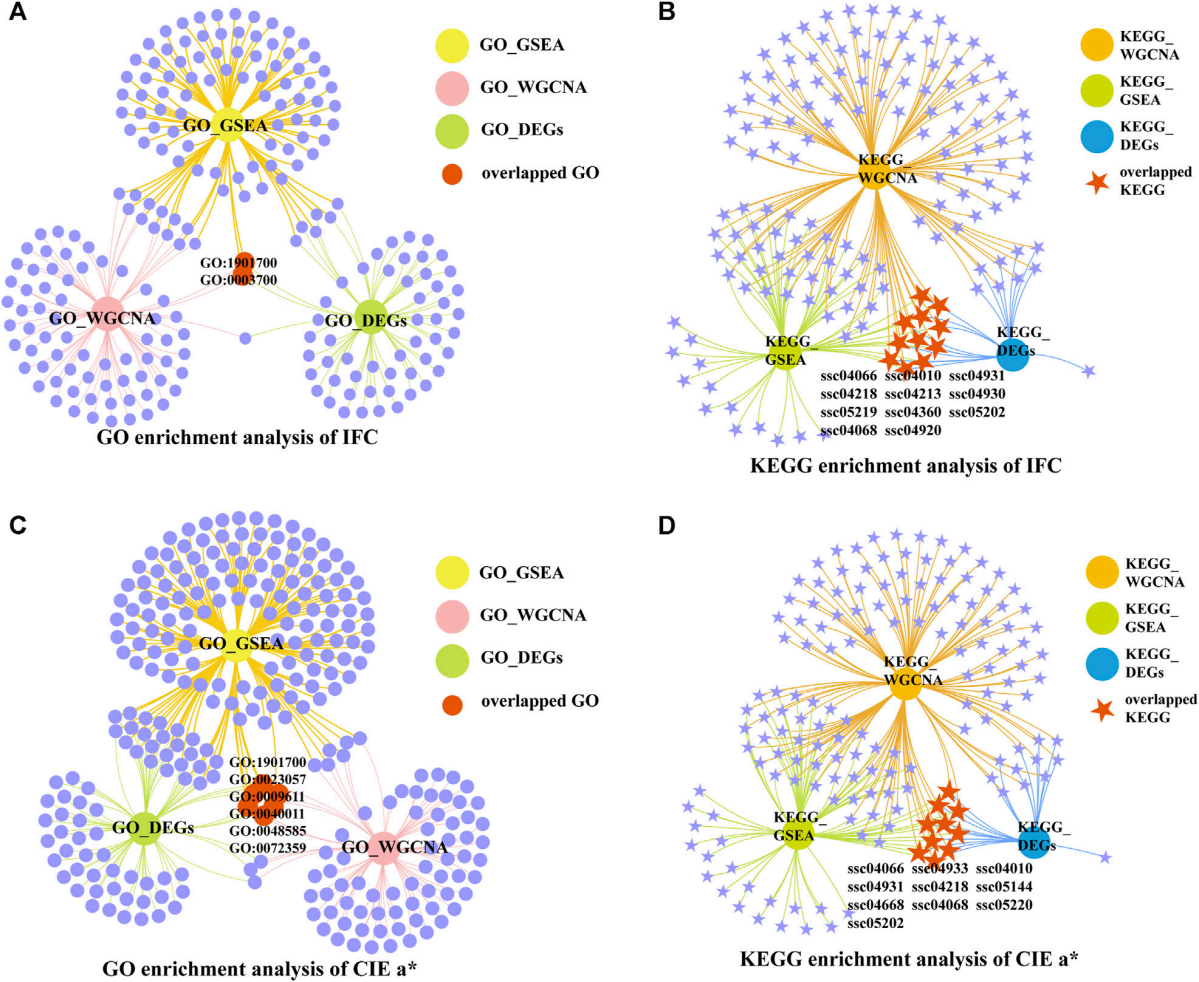
Venn network diagrams of enrichment analysis. **(A)** Venn network diagram of significantly enriched GO terms under three conditions for IFC. **(B)** Venn network diagram of significantly enriched KEGG pathways under three conditions for IFC. **(C)** Venn network diagram of significantly enriched GO terms under three conditions for the CIE a* value. **(D)** Venn network diagram of significantly enriched KEGG pathways under three conditions for CIE a* value.

To determine the candidate genes affecting the IFC and CIE a* value, we first screened the overlapping GO terms and KEGG pathways for each trait based on the functional enrichment analysis results of overlapping DEGs, GSEA, and WGCNA. The DEGs in the overlapping GO terms and KEGG pathways were selected as candidate genes. Finally, hub genes with a connectivity value exceeding ten were obtained by constructinga PPI network of candidate genes. For IFC, 2 overlapping GO terms and 11 overlapping pathways were identified (Figures 6A, B). Most of these overlapping GO terms and pathways were involved in lipid metabolism, such as response to oxygen-containing compounds (GO:1901700), DNA-binding transcription factor activity (GO:0003700), insulin resistance (ssc04931), the MAPK signaling pathway (ssc04010), adipocytokine signaling pathway (ssc04920), the HIF-1 signaling pathway (ssc04066), and the FoxO signaling pathway (ssc04068). For the CIE a* value, 6 overlapping GO terms, and 10 overlapping pathways were identified (Figures 6C, D). Most of these overlapping GO terms, and pathways were involved in oxidative phosphorylation, system development, and lipid metabolism, such as response to oxygen-containing compounds (GO:1901700), negative regulation of signaling (GO:0023057), response to wounding (GO:0009611), circulatory system development (GO:0072359), the FoxO signaling pathway (ssc04068), the adipocytokine signaling pathway (ssc04920), the MAPK signaling pathway (ssc04010), and the HIF-1 signaling pathway (ssc04066).

We selected terms and pathways associated with lipid metabolism and redox in overlapping GO terms and KEGG pathways, and DEGs located in these terms and pathways were considered candidate genes. The selected GO terms and KEGG pathways for the IFC and CIE a* value are shown in Table 2 and Table 3. The results showed that 47 and 53 genes can be considered candidate genes for the IFC and CIE a* value, respectively. These candidate genes were used for subsequent PPI network construction. It was worth noting that among these two traits, there was one GO term (response to oxygenated compounds) and three KEGG pathways (adipocyte cytokine signaling pathway, MAPK signaling pathway, and HIF-1 signaling pathway) that were consistent, and these two traits shared 18 candidate genes (Supplementary Table S15).

**TABLE 2:** Overlapping significantly enriched GO terms based on GO enrichment of DEGs, GSEA, and WGCNA.

Trait	GO ID	Description	GO_DEGs *q* value	GO_GSEA *q* value	GO_WGCNA *q* value	Overlapping DEGs
IFC	GO:1901700	response to oxygen-containing compound	0.069	0.047	0.005	*APOD, INHBB, CEBPB, NR4A3, SOX9, MYOD1, CYP26B1, BGLAP, PANX1, THBS1, PCK1*
	GO:0003700	DNA-binding transcription factor activity	0.078	0.001	0.050	*TGIF1, RUNX1, FOSL2, KLF10, MAFK, SMAD1, MAFF, CSRNP1, CEBPB, NR4A3, SIM1, ATF3, SOX9, MYOD1, CREM, ZSCAN20, KLF5, FOSL1*
CIE a*	GO:1901700	response to oxygen-containing compound	0.023	<0.001	0.005	*THBS1, INHBB, FOXO1, EGR1, PLSCR4, PLK3, CEBPB, SOCS1, SOX9, GJA1, SLC25A33, SLC11A1, NOCT, CCL2, SLC1A1, APOD*
	GO:0023057	negative regulation of signaling	0.034	0.004	0.012	*ADRB2, THBS1, SLC25A5, ADM, SIAH2, SPRY1, SOCS3, INHBB, EGR1, ARRDC3, DUSP5, SOCS1, SOX9, GJA1, APOD*
	GO:0009611	response to wounding	0.043	0.001	0.035	*CCN1, PPL, SERPINE1, THBS1, F3, INHBB, SLC1A1, ITGA5, APOD*
	GO:0072359	circulatory system development	0.043	<0.001	<0.001	*CCN1, SERPINE1, JUNB, THBS1, ADM, VEGFA, TIPARP, ITGA5, ANGPTL4, F3, EGR2, SOX9, GJA1, SLC1A1*

**TABLE 3:** Overlapping significantly enriched KEGG pathways based on KEGG enrichment of DEGs, GSEA, and WGCNA.

Trait	KEGG ID	Description	KEGG_DEGs *q* value	KEGG_GSEA *q* value	KEGG_WGCNA *q* value	Overlapping DEGs
IFC	ssc04931	Insulin resistance	<0.001	0.07	<0.001	*INSR, PPARGC1A, PTPN1, TRIB3, IRS2, PRKAG2, SOCS3, GFPT2*
	ssc04010	MAPK signaling pathway	0.002	0.052	<0.001	*MAP2K3, FLNC, VEGFA, GADD45A, INSR, HSPB1, MAP3K8, GADD45G, MYC, IL1RAP*
	ssc04920	Adipocytokine signaling pathway	0.002	0.071	<0.001	*PPARGC1A, IRS2, PRKAG2, SOCS3*
	ssc04066	HIF-1 signaling pathway	0.006	0.021	<0.001	*VEGFA, INSR, IL6R, SERPINE1, HK3, TIMP1*
	ssc04068	FoxO signaling pathway	0.02	0.029	<0.001	*GADD45A, INSR, GABARAPL1, IRS2, PRKAG2, GADD45G*
CIE a*	ssc04068	FoxO signaling pathway	0.002	<0.001	<0.001	*GADD45A, GABARAPL1, IRS2, PRKAG2, GADD45B, FOXO1, IL6, PLK3, FBXO32*
	ssc04920	Adipocytokine signaling pathway	0.005	0.097	<0.001	*PPARGC1A, IRS2, PRKAG2, CPT1A, SOCS3*
	ssc04010	MAPK signaling pathway	0.043	0.013	<0.001	*GADD45A, FLNC, GADD45B, VEGFA, FGF6, DUSP1, IL1RAP, DUSP4, MYC, DUSP5, DUSP2*
	ssc04066	HIF-1 signaling pathway	0.074	0.021	<0.001	*SERPINE1, VEGFA, TIMP1, IL6, HK2*

### 3.7 Hub genes

**FIGURE 7:**
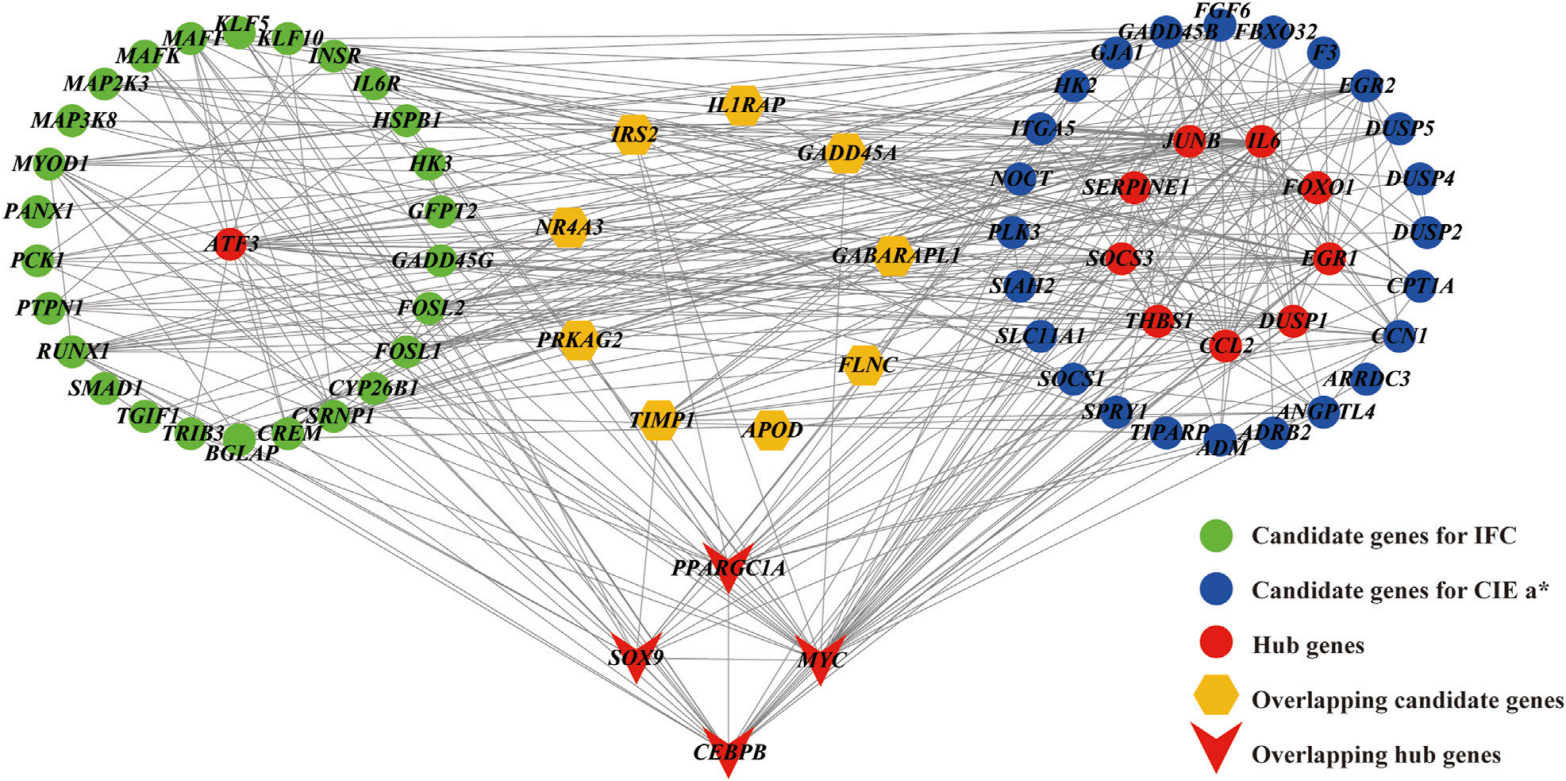
Protein protein interaction (PPI) network for the candidate genes affecting the IFC and CIE a* value. Edges (gray lines) between nodes indicate the interaction of genes in the network. Green circles represent candidate DEGs for IFC, and blue circles represent candidate DEGs for CIE a*. Brown hexagos represent overlapping candidate DEGs for IFC and CIE a*. Red circles and V-shapes represent DEGs with connectivity greater than 10 and are considered hub genes. Hub genes shared by the IFC and CIE a* value are represented by V-shapes.

The interaction relationships of candidate genes affecting the IFC and CIE a* value were obtained by constructing PPI networks (Figure 7). According to the degree of connectivity, five hub genes (*ATF3*, *SOX9*, *PPARGC1A*, *CEBPB*, and *MYC*) with a connectivity value greater than ten were identified as hub genes for IFC trait. Functional enrichment analysis showed that *CEBPB*, *SOX9*, and *PPARGC1A* were mainly involved in the transcriptional regulation of white adipocyte differentiation and the regulation of fatty acid oxidation. For the CIE a* value, 13 hub genes (*IL6*, *MYC*, *EGR1*, *CEBPB*, *JUNB*, *THBS1*, *SERPINE1*, *SOCS3*, *DUSP1*, *SOX9*, *PPARGC1A*, *CCL2*, and *FOXO1*) were identified as hub genes. Functional enrichment analysis showed that *SOCS3*, *IL6*, *FOXO1*, *CEBPB*, *SOX9*, and *PPARGC1A* were mainly involved in the adipocytokine signaling pathway, insulin resistance, FoxO signaling pathway, AMPK signaling pathway, and PI3K-Akt signaling pathway. Notably, *MYC*, *CEBPB*, *SOX9*, and *PPARGC1A* were considered hub genes (transcription factors) affecting both traits, and their expression levels were significantly higher in the low group than in the high group.

## 4 Discussion

DLY pork is dominant in the pork industry; however, its IFC is low, and the meat has a paler color, resulting in limited competitiveness within the premium pork market segment (Chen et al., 2018; Wang et al., 2020). As a result, breeders are eager to undertake genetic improvements in both IFC and redness (CIE a*) meat color concurrently to cater to consumer market demands. In this study, a highly significant positive correlation (*R* = 0.309, *p* < 0.001) between the IFC and the CIE a* value was observed, similar to previous reports by Mortimer et al. (Mortimer et al., 2014) and Zhang et al. (Zhang et al., 2022), in which they discovered the correlation coefficients of the IFC and CIE a* value was 0.260 and 0.323, respectively. The interaction between IFC and meat color is intricate. Several studies have shown that muscles with a higher percentage of red muscle fibers (higher CIE a* values) tend to have a higher IFC (Karlsson et al., 1999; Guo et al., 2011). On the one hand, this is because red muscle fibers contain more neutral fat. On the other hand, the red muscle fiber contains more mitochondria, which are the prominent organelles for fatty acid β-oxidation. Therefore, more lipids may accumulate around the red muscle fibers (internally and externally) to ensure β-oxidation and provide energy to the body. However, the relationship between IFC and muscle redness has not been fully demonstrated. Numerous studies have found significant correlations between IFC and CIE a*, suggesting that there might be similarities in the genetic background regulating changes in both the IFC and redness value. Consequently, transcriptome analysis was conducted using individuls with extreme IFC and CIE a* values to identify hub genes and metabolic pathways co-regulating IFC and the redness of pork.

Conducting transcriptomic analysis based on extreme phenotypes is a commonly employed method to identify key genes influencing target traits. For instance, Wang et al. (2023) in the Anqing Six-end-white pigs, employed RNA-seq on high and low IFC groups to discern critical genes affecting intramuscular fat deposition. Ninety-seven DEGs obtained in their study overlapped with those identified in our high and low IFC groups, including *MYC*, *ATF3*, and *LEP*, which have been reported as candidate genes related to lipid metabolism. Furthermore, Fernández-Barroso et al. (2022) conducted RNA-seq in the LD muscle of Iberian pigs based on extreme phenotypes of myoglobin (CIE a* value). Among the 57 DEGs they obtained, three genes, such as *CCL2*, *VSTM1*, and *ACKR2*, were consistent with our results, and these genes might participate in metabolic pathways linked to redox reactions. Thus, we can conclude that conducting RNA-seq based on extreme phenotypes is an effective strategy.

In this study, WGCNA was used to detect the vital genes and modules associated with the IFC and CIE a* values using transcriptome data from 19 samples. The results of the WGCNA showed that the purple module demonstrated a positive correlation with both th1e IFC and CIE a* value. In contrast, the dark grey, dark red, and black modules exhibited negative correlations with the IFC and CIE a* value. These four modules contained a total of 6,045 genes encoding proteins. Based on the overlap analysis between the DEGs (DEGs of the IFC and DEGs of the CIE a* value) and the WGCNA results, more than 70% of the DEGs could be detected by WGCNA, indicating the similarity between these two analysis methods and further proving the reliability of the results of this study. However, some genes associated with the IFC and CIE a* value identified by WGCNA did not exhibit differential expression in the high and low groups. This observation suggests that WGCNA recognized additional information by establishing interconnected networks between genes, aligning well with the foundational principles of WGCNA. This was consistent with the findings of Xing et al. (2021).

The IFC and CIE a* groups shared four significantly enriched pathways: the FoxO signaling pathway (ssc04068), adipocytokine signaling pathway (ssc04920), MAPK signaling pathway (ssc04010), and HIF-1 signaling pathway (ssc04066) (Table 3). The FoxO signaling pathway governs glucose and lipid metabolism by controlling genes associated with gluconeogenesis, glycogenolysis, and lipid metabolism (Lee and Dong, 2017). It also impacts fatty acid oxidation and storage across diverse tissues (Chen et al., 2023a). Although the direct connection between the FoxO pathway and myoglobin oxidation has not been extensively documented, it is conceivable that this pathway may indirectly influence oxidative processes by regulating energy metabolism and responses to oxidative stress (Egan and Zierath, 2013). The adipocytokine signaling pathway is linked with adipocyte-related functions and metabolism. It modulates insulin sensitivity, glucose uptake, and lipid metabolism, affecting the release of adipokines that influence lipid homeostasis and inflammation (Gu et al., 2019). This pathway likely indirectly affects myoglobin oxidation by influencing factors connected to metabolism and inflammation, thus potentially impacting oxidative processes in muscle tissues (Jorge et al., 2011). The MAPK signaling pathway is integral to various cellular processes, encompassing cell growth, differentiation, and metabolism. It can impact lipid metabolism by regulating genes related to lipogenesis, lipolysis, and fatty acid oxidation (Chen et al., 2023b; Wang et al., 2023). This pathway may contribute to muscle oxidative processes by mediating cellular reactions to stress, lipid peroxidation, and growth cues, thereby influencing myoglobin oxidation under specific conditions (Xu et al., 2018). Activated in response to low oxygen levels, the HIF-1 signaling pathway orchestrates adaptive responses to hypoxia. It influences glycolysis, lipid, and energy metabolism when oxygen levels are low (Zhang et al., 2023). The HIF-1 pathway can affect myoglobin oxidation by regulating the response to hypoxia, potentially influencing oxidative metabolism and the role of myoglobin in oxygen transport and storage (Elkholi et al., 2022). In summary, these pathways may play pivotal roles in both fatty acid metabolism and myoglobin oxidation.

The DEGs in Table 2 and Table 3 were considered candidate genes influencing the IFC and CIE a* values, and the PPI network was constructed based on them (Figure 7). Based on the degree of connectivity, 5 hub genes (*ATF3*, *SOX9*, *PPARGC1A*, *CEBPB*, and *MYC*) with a connectivity value exceeding ten were regarded as hub genes potentially influencing IFC. Similarly, 13 hub genes impacting the CIE a* value were identified, including *IL6*, *MYC*, *EGR1*, *CEBPB*, *JUNB*, *THBS1*, *SERPINE1*, *SOCS3*, *DUSP1*, *SOX9*, *PPARGC1A*, *CCL2*, and *FOXO1*. *ATF3* (activating transcription factor 3), a member of the CREB family of basic leucine zipper transcription factors (TFs). It has been found that the deletion of *ATF3* results in increased lipid body accumulation, and *ATF3* directly regulates transcription of the gene encoding cholesterol 25-hydroxylase (Gold et al., 2012).

*IL6* (interleukin-6) is a pivotal regulatory factor for lipolysis and beta-oxidation. Numerous *in vitro* studies have substantiated that treatment with *IL6* enhances lipolysis and beta-oxidation in both myotubes and adipocytes (Bae et al., 2023; Jackson et al., 2023). *EGR1* (Early growth response 1) is a transcription factor. Mohtar et al. found that insulin/mTORC1-inducible *EGR1* binds to the leptin promoter and activates leptin expression in 3T3-L1 adipocytes, regulating lipid metabolism (Mohtar et al., 2019). The results of Yan et al. suggested that inhibition of *JUNB* might be a key indicator of the regulation of the APOA2-associated PPARα pathway (Yan et al., 2020). *APOA2* is a well-known member of the apolipoprotein family (Ballester et al., 2016), and the PPARα pathway is also a key pathway in regulating lipid metabolism (Cao et al., 2023). *THBS1* (thrombospondin-1) is a prototypical matricellular protein. *THBS1*-null mice exhibited elevated free fatty acids and triglycerides compared to wild-type mice, suggesting impaired fatty acid uptake (Kong et al., 2013). *SERPINE1* (Serpin Family E Member 1), also known as plasminogen activator inhibitor type 1 (PAI-1), is a member of the serine proteinase inhibitor (serpin) superfamily. Several findings have shown that PAI-1 might promote the differentiation of mesenchymal stem cells toward adipogenesis, and PAI-1 deficiency attenuates changes in the levels of adipogenic genes such as PPARγ and aP2 (Tamura et al., 2013; Hu et al., 2019). *SOCS3* (suppressor of cytokine signaling 3) plays an important role in regulating energy metabolism processes. In recent years, researchers have found that *SOCS3* is involved in the AMPK signaling pathway, insulin resistance, adipocytokine signaling pathway, and JAK/STAT pathway, is activated/triggered by leptin signals, and plays important roles in lipid metabolism processes (Liu et al., 2014; Fang et al., 2020; Yang et al., 2020). DUSPs (dual-specificity phosphatases) are the key phosphatases in the MAPK pathway. Recently, *DUSP1* was suggested to play a critical role in the switch from oxidative to glycolytic myofibers (Flach et al., 2011), and can regulate fatty acid oxidation (Roth et al., 2009). *CCL2* (chemokine ligand 2) is a member of the C–C motif family of chemokines. Kang et al. found that after *CCL2* binds to its receptor *CCR2*, it can reduce lipid peroxidation by inhibiting *CCR2*, indicating its important regulatory role in lipid oxidation metabolism (Roth et al., 2009). Current studies suggest that the transcription factor *FOXO1* (forkhead box protein O1) is involved in lipid metabolism and lipolysis in adipocytes (Chakrabarti and Kandror, 2009; Chakrabarti et al., 2011). Song et al. found that interfering with *FOXO1* negatively regulated the expression of adipogenic differentiation marker genes and lipid anabolism marker genes, thus reducing triglyceride content and inhibiting the generation of lipid droplets in bovine adipocytes (Song et al., 2023).

It is worth noting that these two traits share four hub genes: *MYC*, *CEBPB*, *SOX9*, and *PPARGC1A*. *MYC* is a transcription factor that regulates cell proliferation and differentiation in healthy cellular processes. Hall et al. revealed that the activation of *MYC* led to the accumulation of cholesteryl esters stored in lipid droplets (Hall et al., 2020). A previous study found that *MYC* is involved in the MAPK signaling pathway, promoting the glycolysis process in fish T cells (Wei et al., 2020). In addition, *MYC* is involved in the WNT signaling pathway and serves as a target gene/transcriptome factor for WNT, regulating myogenesis (Karczewska-Kupczewska et al., 2016). *CEBPB* (CCAAT/enhancer binding protein β) is a member of the transcription factor family of CEBP. Several studies have reported that *PPARGC1A* (PPAR coactivator-1α, also known as *PGC1α*), a transcriptional co-activator of PPARγ, can bind to *CEBPB* and form a transcription complex. This complex may promote the transcription of *CPT1A* (carnitine palmitoyl transferase 1 A) and activate fatty acid β-oxidation (Du et al., 2019; Wu et al., 2020). *SOX9* (Sex-determining region Y-type box-9) is a member of the Sox supergene family and has been proven to be an essential transcription factor in cartilage formation during chondrocyte proliferation (Akiyama, 2008). Wang et al. confirmed that *SOX9* can directly bind to the promoters of *CEBPB* and *CEBPD*, inhibit their promoter activity, and prevent adipocyte differentiation (Wang and Sul, 2009). This evidence indicated that the *SOX9*/*CEBPB*/*PPARGC1A* axis might play an essential regulatory role in fatty acid β-oxidation. Myoglobin is an oxygen-binding hemeprotein generally localized to oxidative muscle and functions as an oxygen store and reactive oxygen species scavenger (Gödecke, 2010). Schlater et al. confirmed that an increase in lipids could stimulated an increase in myoglobin content in muscle cells of C2C12 mice, which was closely related to fatty acid beta oxidation (Schlater et al., 2014). In summary, we speculated that the *SOX9*/*CEBPB*/*PPARGC1A* axis plays a vital role in the co-regulation of IFC deposition and changes in the redness of meat color. The expression levels of the upstream gene *STAT3* (signal transducer and activator of transcription 3) and downstream *CPT1A* genes (log2FC = 1.17) in the *SOX9*/*CEBPB*/*PPARGC1A* axis were also significantly different in the high and low groups in this study, further supporting the importance of this pathway in the synergistic regulation of lipid and myoglobin metabolism. Thererore, it will be particularly interesting to investigate the co-regulatory mechanism of the *SOX9*/*CEBPB*/*PPARGC1A* axis in IFC and CIE a* value traits in further studies.

## 5 Conclusion

In this study, we identified 5 hub genes influencing the IFC and 13 hub genes affecting the CIE a* value through integrating differential gene expression analysis, WGCNA, functional enrichment under various conditions, and PPI network analysis. These genes maninly participate in multiple lipid and myoglobin metabolism pathways. Moreover, we discovered that the *SOX9*/*CEBPB*/*PPARGC1A* axis is the potential pathway co-regulating lipid deposition and the myoglobin redox reaction. These hub genes and the *SOX9*/*CEBPB*/*PPARGC1A* axis may be critical for the IFC and CIE a* value; however, the functions and regulatory mechanism of these hub genes, particularly the *SOX9*/*CEBPB*/*PPARGC1A* axis, still need to be further elucidated.

## Data Availability

The original contributions presented in the study are publicly available. This data can be found in the NCBI database, under BioProject PRJNA1052206, https://www.ncbi.nlm.nih.gov/bioproject/1052206.
