# Supplementary material for: Construction of a co-expression network affecting intramuscular fat content and meat color redness based on transcriptome analysis
Source: Front Genet. 2024 Feb 13;15:1351429. doi: 10.3389/fgene.2024.1351429 (PMC10897757; doi:10.3389/fgene.2024.1351429)
Supplement: Supplementary file 2 [file DataSheet1.docx]

***Supplementary Material***

# Supplementary Figures and Tables

## 1.1 Supplementary Figures


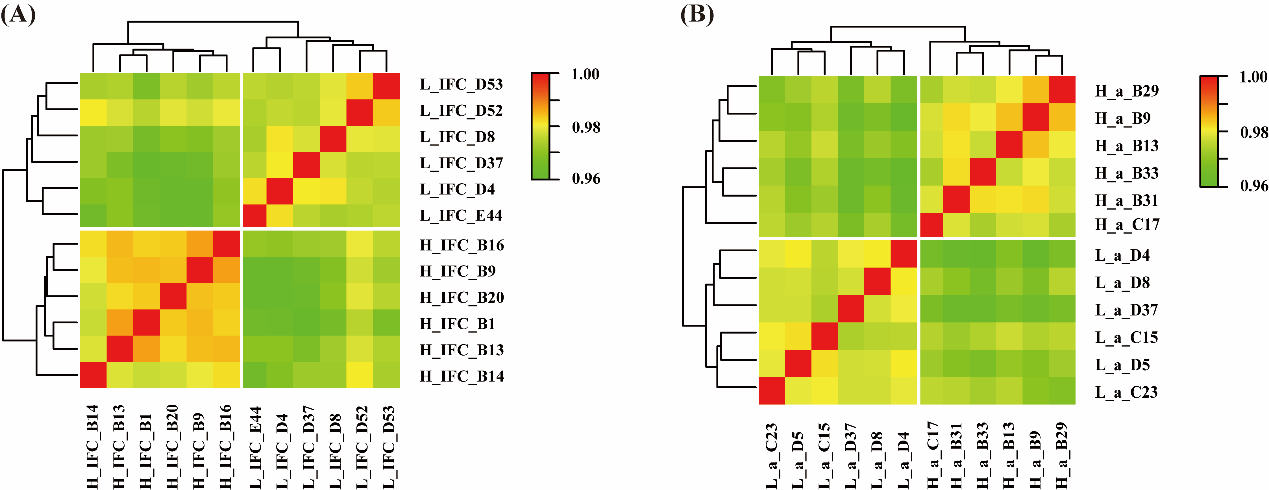


**Supplementary Figure 1.** (A) heatmap of clustering of samples in the high and low intramscular fat content (IFC) groups. (B) heatmap of clustering of samples in the high and low CIE a* values groups.


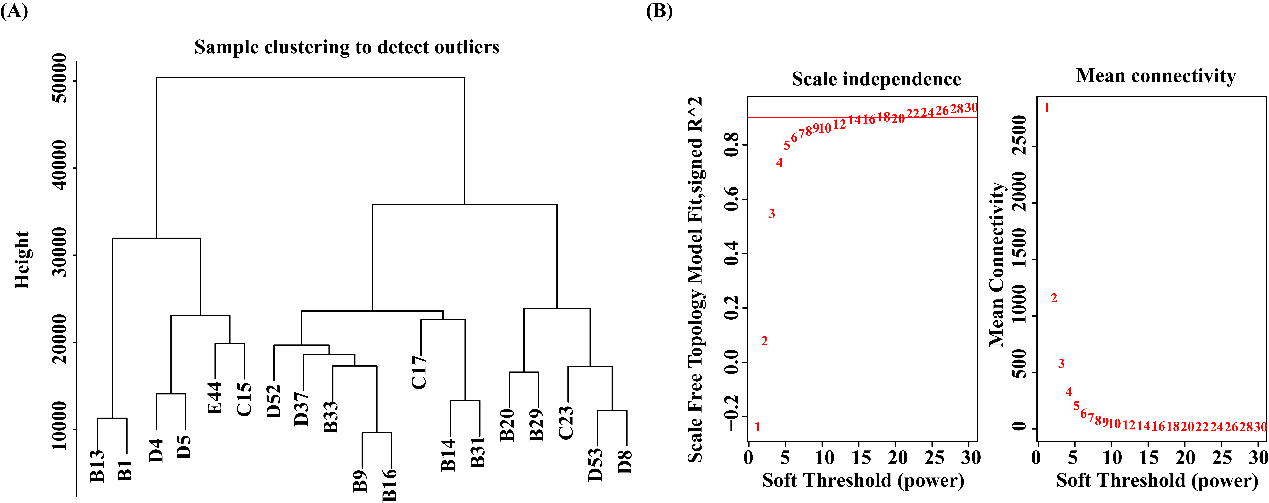


**Supplementary Figure 2.** Sample clustering and soft thresholding powers. (A) Hierarchical cluster analysis revealed no outliers among 19 samples. (B) Analysis of free‑scale network topology for different soft‑thresholding powers.


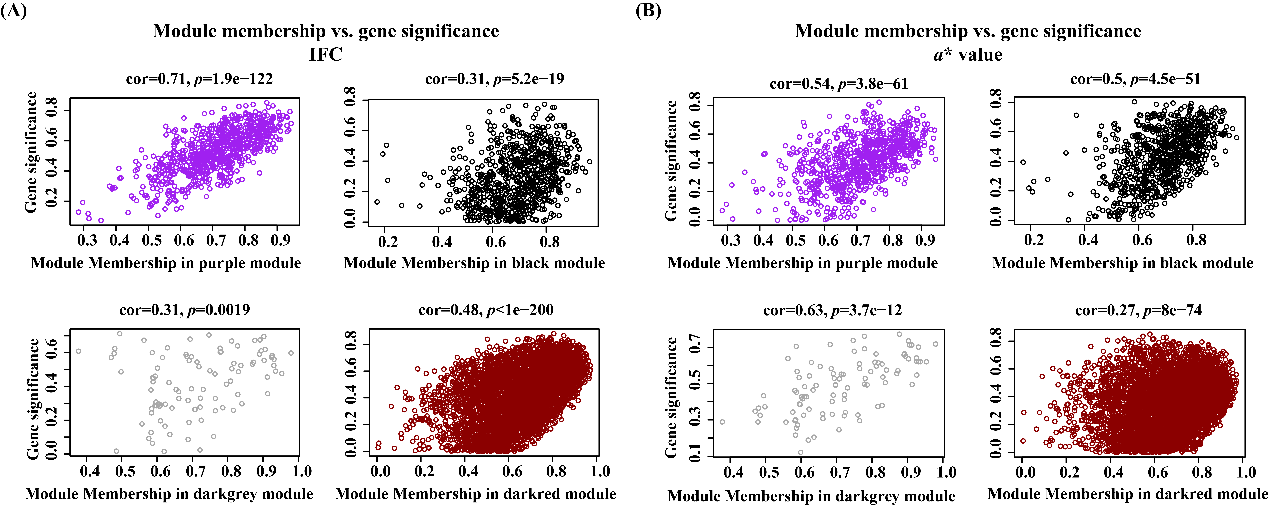


**Supplementary Figure 3.** Scatterplot of Gene Significance (GS) for IFC and CIE a* value vs. Module Membership (MM) in purple, black, darkgrey and darkred modules. (A) and (B) represent IFC and CIE a* value, respectively.


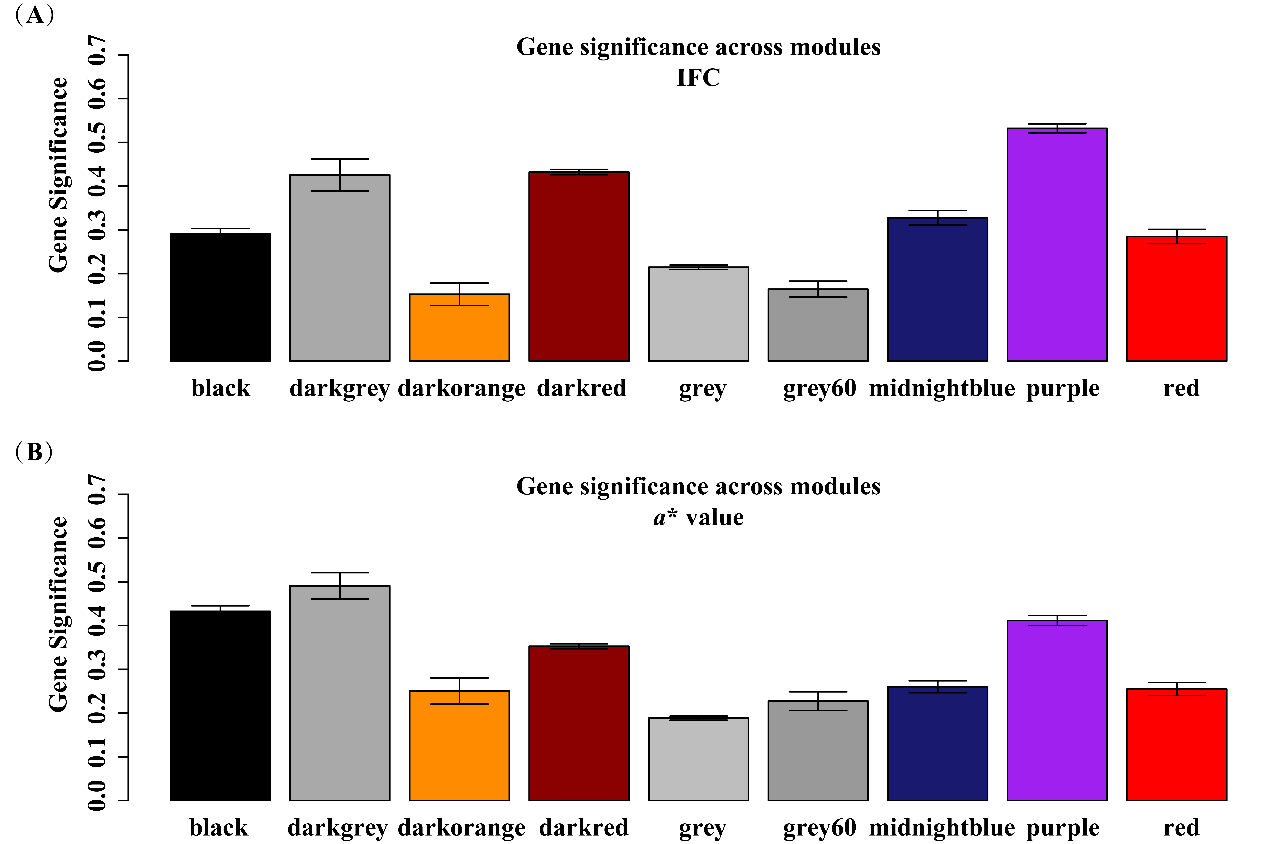


**Supplementary Figure 3.** The absolute correlation between the genes in each module. (A) and (B) represent IFC and CIE a* value, respectively.
